# Involvement of the SIRT1-NLRP3 pathway in the inflammatory response

**DOI:** 10.1186/s12964-023-01177-2

**Published:** 2023-07-28

**Authors:** Huiyue Chen, Jiayu Deng, Huan Gao, Yanqing Song, Yueming Zhang, Jingmeng Sun, Jinghui Zhai

**Affiliations:** 1 Department of Clinical Pharmacy, the First Hospital of Jilin University, Changchun, , Jilin China; 2 School of Pharmaceutical Science, Jilin University, Changchun, Jilin China; 3 Department of Pharmacy, Lequn Branch, the First Hospital of Jilin University, Changchun, Jilin China

**Keywords:** Sirtuin 1, NACHT, LRR and PYD domains-containing protein 3, Inflammatory response, Nuclear factor erythroid 2-related factor 2, Nuclear factor-kappa B

## Abstract

The silent information regulator 2 homolog 1-NACHT, LRR and PYD domains-containing protein 3 (SIRT1-NLRP3) pathway has a crucial role in regulation of the inflammatory response, and is closely related to the occurrence and development of several inflammation-related diseases. NLRP3 is activated to produce the NLRP3 inflammasome, which leads to activation of caspase-1 and cleavage of pro-interleukin (IL)-1β and pro-IL-18 to their active forms: IL-1β and IL-18, respectively. They are proinflammatory cytokines which then cause an inflammatory response.SIRT1 can inhibit this inflammatory response through nuclear factor erythroid 2-related factor 2 and nuclear factor-kappa B pathways. This review article focuses mainly on how the SIRT1-NLRP3 pathway influences the inflammatory response and its relationship with melatonin, traumatic brain injury, neuroinflammation, depression, atherosclerosis, and liver damage.

Video Abstract

**Supplementary Information:**

The online version contains supplementary material available at 10.1186/s12964-023-01177-2.

## Background

The inflammatory response is the cause of several diseases, so studying the mechanism of the inflammatory response is a strategy for finding a cure for diseases. Finding appropriate means to control inflammation has always been a “hotspot” in research. Comprehensive understanding of the pathways and molecules involved in inflammation may provide essential information for innovative therapeutic targets. Such pathways and molecules include tumor necrosis factor (TNF)-α, interferon (IFN)-γ, interleukin (IL)-1β, and IL18.

NACHT, LRR and PYD domains-containing protein 3 (NLRP3) is an intracellular sensor that detects a broad range of microbial motifs, endogenous danger signals, and environmental irritants, resulting in the formation and activation of the NLRP3 inflammasome [1]. Refinement of understanding of its activation is continuing, but targeting of it as a therapeutic for multiple diseases is progressing rapidly. Treatment for diseases in which NLRP3 inflammasome is involved has focused on inhibition of the inflammasome-derived cytokine IL-1β [2].

The NLRP3 inflammasome associates with the tubulin cytoskeleton and localizes to mitochondria, where reactive oxygen species (ROS) lead to activation of the NLRP3 inflammasome [3]. The latter mediates caspase-1 activation and secretion of the pro-inflammatory cytokines IL-1β/IL-18 in response to microbial infection and cellular damage. Active caspase-1 cleaves the cytokines pro-interleukin-1β (pro-IL-1β) and pro-IL-18 into their mature and biologically active forms [4–6]. IL-1β induces the expression of genes that control fever, the pain threshold, vasodilatation, and hypotension, and its reception leads to an endothelial-cell response that facilitates the infiltration of immune cells to infected or damaged tissues [7]. IL-18 is necessary for IFN-γ production and is a co-stimulatory cytokine that mediates adaptive immunity [7]. However, aberrant activation of the NLRP3 inflammasome has been linked with several inflammatory disorders: cryopyrin-associated periodic syndromes, Alzheimer’s disease, diabetes mellitus, prion diseases, and atherosclerosis [8]. Assembly of the NLRP3 inflammasome leads to caspase-1-dependent release of the pro-inflammatory cytokines, IL-1β and IL-18, as well as to gasdermin D-mediated pyroptotic cell death [9]. Understanding the mechanisms of activation of the NLRP3 inflammasome could enable the development of its specific inhibitors to treat NLRP3 inflammasome-related diseases.

Silent information regulator 2 homolog 1(SIRT1) is a member of the nicotinamide adenine dinucleotide (NAD)-dependent sirtuin family. SIRT1 activates the deacetylation of acetyl groups on the lysine residues of proteins, thereby regulating their functions [10, 11]. SIRT1 helps to protect against multiple oxidative inflammatory injuries via induction of antioxidant defense pathways and suppression of the inflammatory response [12, 13]. Some studies have demonstrated that expression of pro-inflammatory cytokines is inhibited by SIRT1 because it can mediate the initiation and progression of inflammation (e.g., deacetylating nuclear factor kappa B (NF-κB)) [14, 15]. SIRT1 regulates several cellular processes: aging, metabolism, redox homeostasis, survival, and inflammation [16]. SIRT1 deacetylates various target genes, including those of histone proteins, p53, and NF-κB, and regulates their activities [17]. SIRT1 activation positively modulates nuclear factor erythroid 2-related factor 2 (Nrf2) antioxidant signaling and negatively modulates the transcriptional activity of NF-κB p65 and its downstream inflammatory cascade [18, 19].

Activation of the toll-like receptor (TLR) 4/NF-κB signaling pathway results in activation of inducible NLRP3 and an increase in constitutive expression of pro-IL-1β and pro-IL-18 [17]. Nrf2 activates transcription of several hundred genes encoding anti-oxidant detoxification as well as the enzymes involved in the metabolism of iron, lipids, and glucose. Anti-oxidant enzymes mediate the synthesis and transport of non-enzymatic anti-oxidant-defense molecules (mainly glutathione) [20]. The anti-oxidant, anti-inflammatory, and cytoprotective effects of Nrf2 whose one of the anti-inflammatory mechanisms is the competition with NF-κB for DNA binding [21, 22] have been shown in various studies [23]. Heme oxidase (HO)-1 is an important target of it and also shows an anti-inflammatory effect by inhibiting NF-κB expression [24].

The mechanisms contributing to anti-inflammatory effects are manifold. They comprise various pathways of secondary signaling-prevention of activation of the NLRP3 inflammasome, inhibition of NF-κB activation, and upregulation of Nrf2 expression [25], but they are important for the treatment of inflammatory response-related diseases. In this review article, we discuss these mechanisms (Table 1).

**Table 1:** The anti-inflammatory pathways of the inflammatory response-related diseases

Organ	Diseases	Pathway of the inhibition	Ref
Brain	Traumatic brain injury Depression Neuroinflammation	SIRT1-NLRP3/ROS SIRT1-NLRP3/NF-κB SIRT1-NLRP3/Nrf2	[26–28] [29] [30]
Heart	Atherosclerosis	SIRT1-NLRP3/IL-1β	[31, 32]
Lung	Chronic obstructive pulmonary disease	SIRT1-NLRP3/IL-1β	[33]
Liver	Liver damage	SIRT1-NLRP3/Nrf2	[34–36]
Pancreas	Diabetes mellitus	SIRT1-NLRP3	[37]

*SIRT1* Silent information regulator 2 homolog 1, *NLRP3*, *NACHT* LRR and PYD domains-containing protein 3, *ROS* Reactive oxygen species, *NF-κB* Nuclear factor-kappa B, *Nrf2* Nuclear factor erythroid 2-related factor 2, *IL-1β* Interleukin (IL)-1β

### Mechanisms of the SIRT1-NLRP3 pathway

**Fig. 1:**
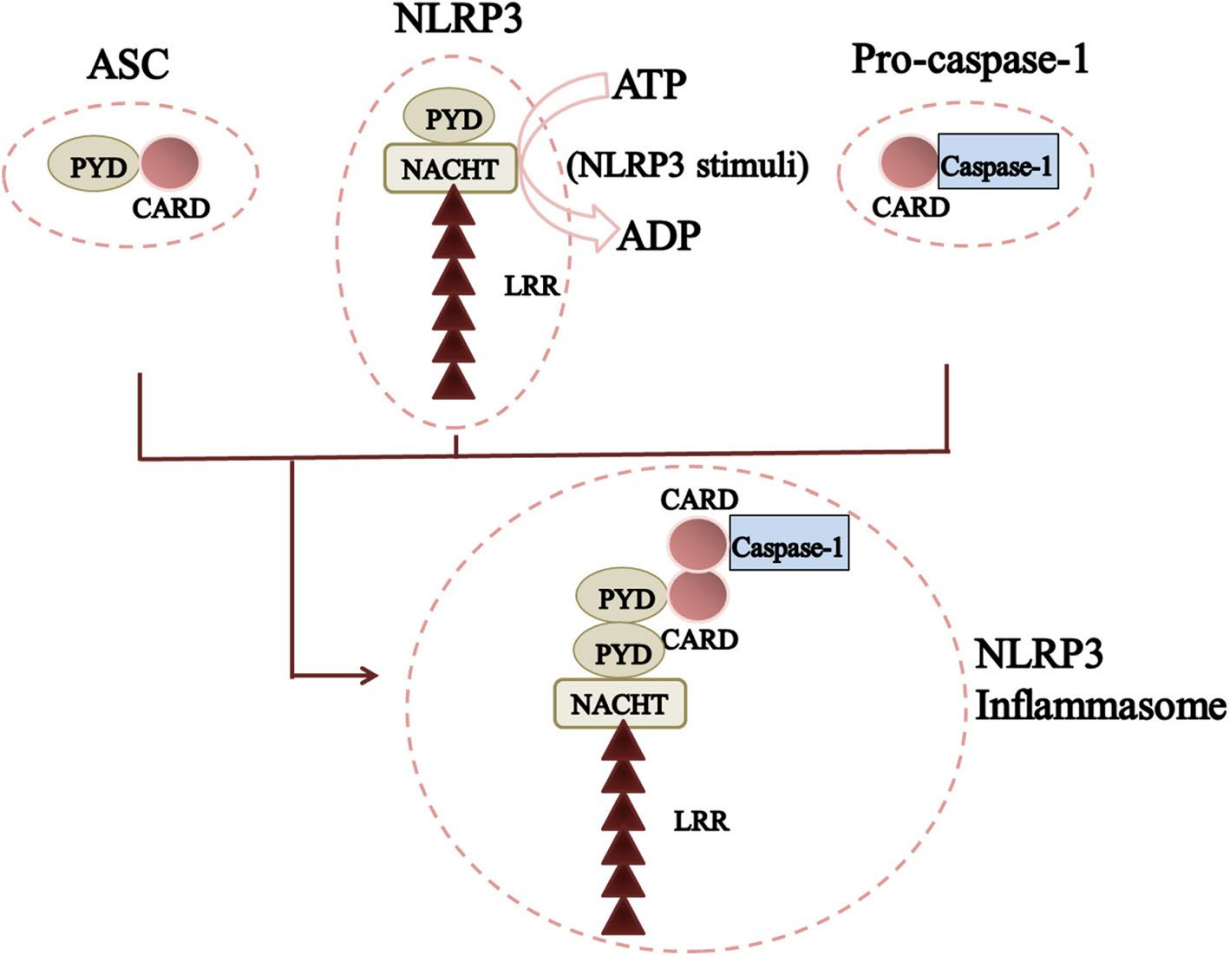
The structure of NLRP3 inflammasome. NLRP3 is a tripartite protein that contains an aminoterminal pyrin domain (PYD), a central NACHT domain, and a carboxy-terminal leucine-rich repeat (LRR) domain. NLRP3 recruits ASCs through PYD-PYD interactions. In turn, pro-caspase-1 is recruited by ASC through CARD-CARD interactions to form the NLRP3-ASC-pro-caspase-1 inflammasome. (NLRP3: NACHT, LRR and PYD domains-containing protein 3; PYD: Pyrin domain; NACHT: Domain present in NAIP, CIITA, HETE and TP1; LRR: Leucine-rich repeat; ASC: a protein; CARD: C-terminal caspase-recruitment domain; ATP: Adenosine triphosphate; ADP: Adenosine diphosphate)

The NLRP3 inflammasome consists of a sensor (NLRP3), an adaptor (ASC; also known as PYCARD), and an effector (caspase-1). NLRP3 is a tripartite protein that contains an aminoterminal pyrin domain (PYD), a central NACHT domain (domain present in NAIP, CIITA, HETE and TP1), and a carboxy-terminal leucine-rich repeat (LRR) domain [G] (Fig. 1). The NACHT domain has adenosine triphosphate (ATP) activity that is vital for the self-association and function of NLRP3 [38]. The LRR domain is thought to induce auto-inhibition by folding-back onto the NACHT domain. The adaptor ASC has two protein-interaction domains: an N-terminal PYD and a C-terminal caspase-recruitment domain (CARD). Full-length caspase-1 has an N-terminal CARD, a central large catalytic domain (p20), and a C-terminal, small-catalytic-subunit domain (p10). Upon stimulation, NLRP3 oligomerizes through homotypic interactions between NACHT domains. Oligomerized NLRP3 recruits ASC through homotypic PYD–PYD interactions and nucleates formation of helical ASC filaments, also through PYD–PYD interactions. Multiple ASC filaments coalesce into a single macromolecular focus, known as an “ASC speck” [39–41]. The assembled ASC recruits caspase-1 through CARD–CARD interactions, and enables proximity-induced self-cleavage and activation of caspase-1. Caspase-1 clustered on ASC self-cleaves at the linker between p20 and p10 to generate a complex of p33 (comprising CARD and p20) and p10, which remains bound to ASC and is proteolytically active [42]. Further processing between the CARD and p20 releases p20-p10 from ASC. The released p20-p10 heterotetramer is unstable in cells, so its protease activity is terminated. Recently, NIMA-related kinase 7 (NEK7), a serinethreonine kinase involved in mitosis, was found to be essential for activation of the NLRP3 inflammasome [43–45]. NEK7 interacts specifically with NLRP3, but not the other inflammasome sensors, nucleotide-binding oligomerization domain, leucine-rich repeat, and caspase recruitment domain-containing 4 (NLRC4) or interferon-inducible protein human Absent in Melanoma 2 (AIM2) [G]. Upon activation of the NLRP3 inflammasome, the NEK7–NLRP3 interaction increases, and NEK7 oligomerizes with NLRP3 into a complex that is essential for formation of the ASC speck and caspase-1 activation [44, 45]. Thus, NEK7 appears to be a core component specific to the NLRP3 inflammasome.

**Fig. 2:**
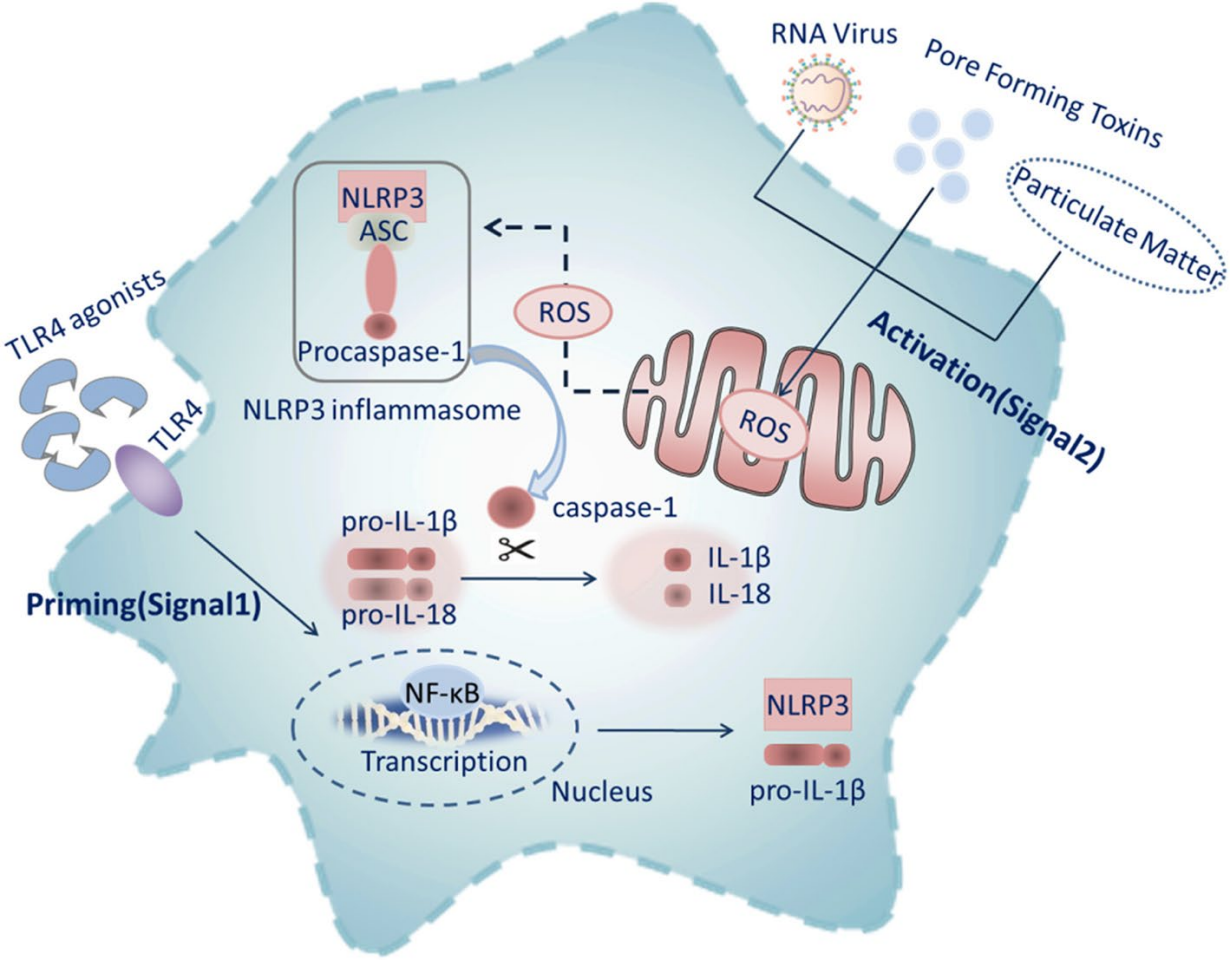
The activation of NLRP3 inflammasome. The priming signal (signal 1) is provided by microbial components or endogenous cytokines, leading to the activation of the transcription factor NF-κB and subsequent upregulation of NLRP3 and pro-IL-1β. The activating signal (signal 2) is from RNA virus, pore-forming toxins, or particulate matter activates the NLRP3 inflammasome with the help of ROS as a by-product of oxidative phosphorylation from mitochondria. (RNA: Ribonucleic acid; TLR: Toll-like receptor; Sirt1: Silent information regulator 2 homolog 1; ROS: Reactive oxygen species; NLRP3: NACHT, LRR and PYD domains-containing protein 3; ASC: a protein; IL: Interleukin; NF-κB: Nuclear factor kappa B)

Activation of the NLRP3 inflammasome occurs mainly in macrophages and microglia [46]. Activation of the NLRP3 inflammasome in macrophages requires two steps: priming and activation. The priming step (signal 1) is provided by inflammatory stimuli such as TLR4 agonists, which induce expression of NF-κB-mediated NLRP3 and pro-IL-1β.The activation step (signal 2) is triggered by pathogen-associated molecular patterns and damage-associated molecular patterns, thereby promoting assembly of the NLRP3 inflammasome and caspase-1-mediated secretion and pyroptosis of IL-1β and IL-18 [47] (Fig. 2). Mitochondrial dysfunction, and release of mtROS into the cytosol, are additional key upstream events implicated in NLRP3 activation. Mitochondria continually produce ROS as a by-product of oxidative phosphorylation, although during cellular stress mtROS levels are greatly increased. Mitophagy [G] is therefore an important regulator of NLRP3 activation as it removes damaged and dysfunctional mitochondria and reduces mtROS. However, the priming step is sufficient for human monocytes to mediate caspase-1 activation and IL-1β release [48]. The activated NLRP3 inflammasome causes the hydrolysis of the inactive procaspase-1 protein, which is then cleaved into active caspase-1. The latter converts IL-1β and IL-18 precursor proteins into mature IL-1β and IL-18 [49]. Then IL-1β and IL-18 cause the inflammatory response.

SIRT1 activation positively modulates Nrf2 antioxidant signaling and negatively modulates the transcriptional activity of NF-κB p65 and its downstream inflammatory cascade [18, 19], which has an anti-inflammatory effect. Moreover, SIRT1 impacts inflammation directly by deacetylating and inactivating the p65 subunit of NF-κB, thereby limiting the expression of NF-κB-dependent pro-inflammatory genes [50]. This may become a hot research direction in the treatment of inflammation-related diseases. This review article focuses mainly on how the SIRT1-NLRP3 pathway influences the inflammatory response.

## SIRT1-NLRP3 pathway in diseases

### SIRT1-NLRP3 pathway and melatonin

#### Melatonin

Melatonin is a hormone mainly produced by the pineal gland. It contributes to the regulation of physiological activities, such as sleep, circadian rhythm, and neuroendocrine processes [51]. Melatonin has anti-inflammation, anti-oxidation, anti-apoptosis, immunomodulatory, and antitumor activities [52, 53]. Thanks to these properties, melatonin has therapeutic benefits for various types of respiratory disease [54, 55].

By inhibiting NLRP3 expression, melatonin diminishes inflammation and influences various molecular pathways involving SIRT1, microRNA, long non-coding RNA, and wingless type (Wnt)/β-catenin [51]. It also activates processes in an anti-inflammatory network in which SIRT1 activation, upregulation of Nrf2 expression, downregulation of NF-κB expression, and release of the anti-inflammatory cytokines IL-4 and IL-10 are involved [56]. In addition, melatonin inhibits pyroptosis as well as the production of mitochondrial and cytosolic ROS and NF-κB signaling. The beneficial effects of melatonin on activation of the NLRP3 inflammasome are associated with activation of Nrf2 and SIRT1, which can be reversed by treatment with Nrf2 siRNA and SIRT1 inhibitors [17].

SIRT1 deacetylates high mobility group box (HMGB)1 [57, 58] to inhibit its nucleocytoplasmic transfer and to prevent its release [59–65]. Importantly, HMGB1 also favors the polarization of macrophages and microglia towards the pro-inflammatory M1 type [66–70]. In fact, melatonin is capable of shifting this balance toward the anti-inflammatory side by favoring M2 and disfavoring M1 polarization, as recently reviewed. With regard to the melatonin–SIRT1 relationship, anti-inflammatory actions via inhibition of HMGB1 expression have been also reported for melatonin [25]. SIRT1 may mediate the effects of melatonin.

#### Melatonin in chronic obstructive pulmonary disease (COPD)

Chronic airway inflammation is a characteristic feature of COPD. Studies have demonstrated that melatonin had a protective effect against COPD. Interestingly, melatonin ameliorates airway inflammation in rats with COPD through stimulation of SIRT1 and subsequent inhibition of the NLRP3 inflammasome [33]. This occurs because SIRT1 is responsible for inhibiting inflammation and respiratory stress by downregulating expression of the NLRP3 inflammasome [71, 72]. In a rat model of COPD, the beneficial effects of melatonin included reduced formation of the NLRP3 inflammasome and IL-1β levels. Those results demonstrated that melatonin prevented COPD development, which was attributed to inhibition of airway inflammation by attenuating expression of the NLRP3 inflammasome and IL-1β [33].

**Fig. 3:**
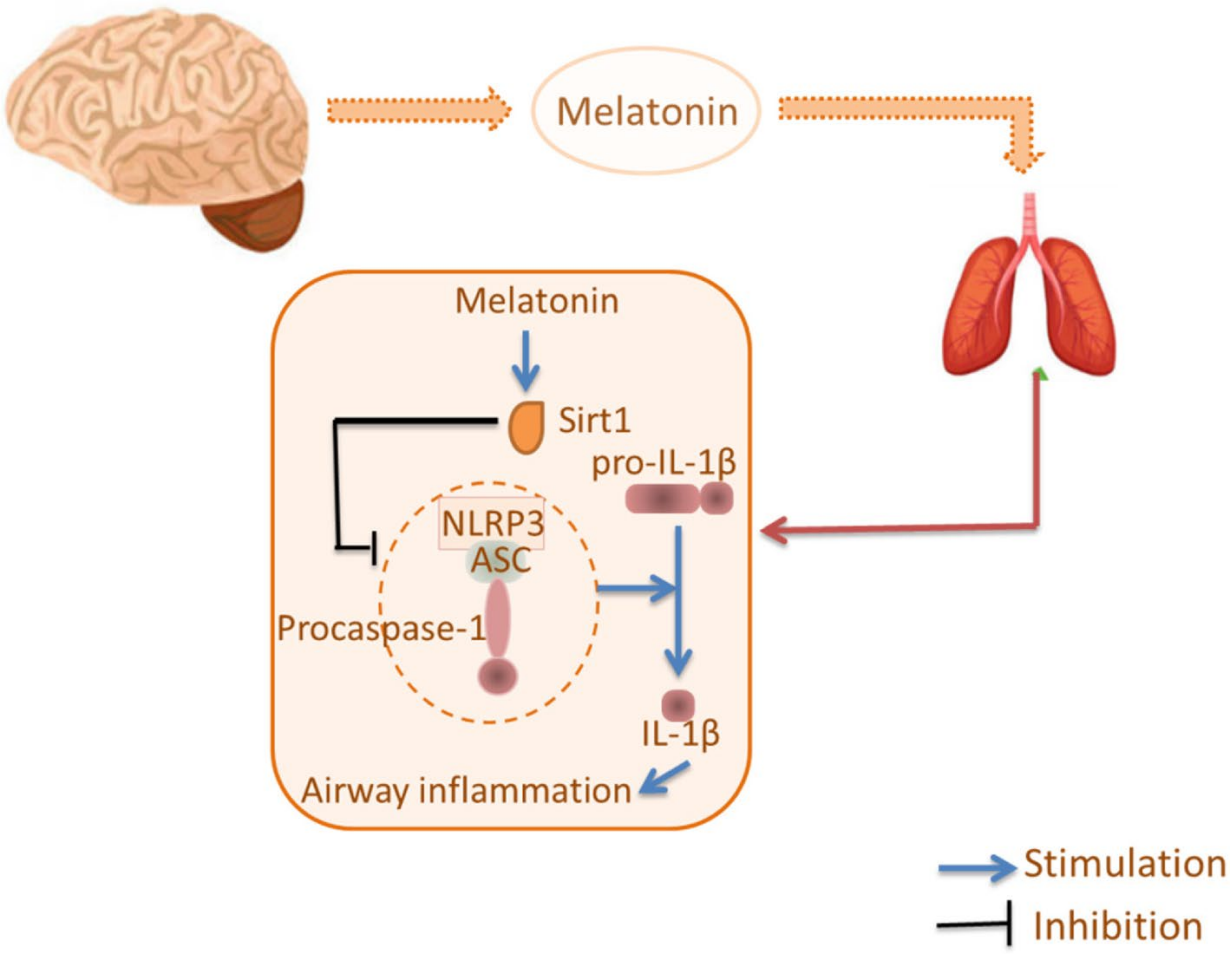
Melatonin in airway inflammation. Melatonin exerted a protective effect which was further suggested to be dependent on targeting its membrane receptor (MT) 1 or 2 against airway inflammation in COPD. This is attributed to the inhibition of NLRP3 inflammasome and IL-1β via the activation of SIRT1 in the lung tissues of whom with COPD. (Sirt1: Silent information regulator 2 homolog 1; NLRP3: NACHT, LRR and PYD domains-containing protein 3; ASC: a protein; IL: Interleukin; MT: Membrane receptor)

Recently, several studies have shown that the NLRP3 inflammasome and IL-1β are involved in the airway inflammation observed in COPD. The latter is characterized by an enhanced inflammatory response in the airway and lung parenchyma [73] and plays an important part in COPD pathogenesis [74, 75]. Several studies have reported that the NLRP3 inflammasome and IL-1β signaling pathway are pivotal in the initiation and persistence of airway inflammation (Fig. 3), which contributes to COPD development [76]. Therefore, the NLRP3 inflammasome and IL-1β have been targets for treatment of COPD. In addition, melatonin has been shown to attenuate the protein expression of NLRP3, cleaved caspase-1, and ASC significantly in lung tissues as compared with that in a COPD group. Those results indicate that melatonin can attenuate airway inflammation with COPD by suppressing the NLRP3 inflammasome and IL-1β signaling pathway (Table 1).

It has been reported that SIRT1 expression is decreased in COPD [77] and that SIRT1 activation inhibits the abnormal inflammatory response in COPD [78, 79]. Recently, melatonin has been reported to promote SIRT1 expression in several conditions [80, 81]. In the present study, some found that melatonin increased the expression of SIRT1 in lung tissues of rats with COPD, while inhibition of SIRT1 by EX527 abolished the protective effect of melatonin against COPD, exhibiting the deteriorated lung function, the increased inflammatory cells and IL-1β level.

The protective effect of melatonin has been attributed to inhibition of the NLRP3 inflammasome and IL-1β signaling pathway via SIRT1 activation in lung tissues afflicted by COPD.

#### Melatonin in aging and various age-related diseases

Aging and various age-related diseases are associated with reductions in melatonin secretion, pro-inflammatory changes in the immune system, a deteriorating circadian system, and reductions in SIRT1 activity. In non-tumor cells, several effects of melatonin are abolished by inhibiting SIRT1 expression, which indicates mediation by SIRT1 [56].

The inclusion of SIRT1 (and perhaps, other sirtuins) into the spectrum of actions of melatonin represents an important step forward in understanding of its aging- and inflammation-related properties. However, one cannot expect that all actions of SIRT1 will mediate melatonergic regulation. SIRT1 is also controlled by various other factors, including accessory components of circadian oscillators that regulate nicotinamide phosphoribosyltransferase (NAMPT) expression and NAD + levels, such as triiodothyronine and glucocorticoids [56]. More extensive studies on melatonin, SIRT1, and microRNAs could reveal additional cases related to the immune system, aging, and age-related diseases.

#### Melatonin in diabetes mellitus

In cultured keratinocytes, exposure to increased glucose concentrations can cause activation of the NLRP3 inflammasome, which is inhibited by melatonin (Table 1). NLRP3 inflammasome has been proposed to sense and mediate downstream inflammatory events of “glucotoxicity” during pathogenesis of type 2 diabetes and thus is responsible for a constant pro-inflammatory status. The present study found that melatonin inhibited pro-inflammatory cytokine levels and NLRP3 inflammasome activation in keratinocytes under high glucose condition, suggesting that melatonin exerts anti-inflammatory effect during diabetic wound healing. Those findings were interpreted as a means for promoting wound healing in people suffering from diabetes mellitus [37].

#### Melatonin in osteogenesis

Knockdown of NLRP3 expression has been reported to attenuate the inhibition of osteogenesis in mice, and similar results have been obtained with melatonin, findings that were interpreted in terms of Wnt/β-catenin signaling. Additional data supporting involvement of inhibition of the NLRP3 inflammasome in the osteogenic action of melatonin were deduced from a counteraction by an activator of the NLRP3 inflammasome: monosodium urate [82]. The role of melatonin in the balance of osteogenic differentiation and osteoclastic activity seems to be very complex and may require consideration of the role of SIRT1 [83–86]. Hence, further studies may be necessary to clarify the relative contributions of the effects of melatonin. With respect to signaling, inhibition of NF-κB activation by melatonin [87] has also been implicated in suppression of the NLRP3 inflammasome [88–92].

#### Melatonin in depression

Melatonin suppresses NLRP3 expression and IL-1β cleavage in the hippocampus. It has been reported that melatonin increases SIRT1 expression in the central nervous system (CNS) and protects the brain in different experimental conditions by activating the SIRT1/Nrf2 signaling pathway [93]. SIRT1 shows wide expression in the CNS, is involved in maintenance of physiological brain functions, and exhibits neuroprotective and anti-inflammatory effects in many neurodegenerative diseases. In addition, melatonin has been shown to inhibit activation of the NLRP3 inflammasome and pyroptosis in murine microglia by activating the SIRT1/Nrf2 signaling pathway [17]. It has been concluded that Nrf2 and SIRT1 signaling pathways are important signaling cascades involved in the preventative and treatment-based effects of melatonin on activation of the NLRP3 inflammasome in microglia [17].

### SIRT1-NLRP3 pathway in traumatic brain injury (TBI)

The inflammatory response in the cerebral cortex has an important role in the progression of secondary injury following TBI. Activation of NLRP3, caspase-1, and SIRT1 has been shown to enhance the production of pro-inflammatory cytokines and ROS following TBI. Activation of the NLRP3 inflammasome and the subsequent inflammatory response in the cerebral cortex are involved in TBI [94–96]. Increasing evidence indicates that the NLRP3 inflammasome participates in the development of CNS disorders such as cerebral ischemia–reperfusion injury [97], neurodegenerative diseases [98], and cerebral tumors [99]. It has been reported that NLRP3 also participates in TBI pathogenesis [100].

Various exogenous and endogenous molecular patterns can activate the NLRP3 inflammasome. ROS have been regarded as important activators of the NLRP3 inflammasome in cardiac ischemia–reperfusion injury [26] and sepsis-induced acute lung injury [27] (Table 1). Meanwhile, SIRT1 is an important regulator of oxidative stress [28].

Enhanced expression of SIRT1 has been reported to have a neuroprotective effect in CNS diseases [101]. Furthermore, studies have shown SIRT1 to be an endogenous protective molecule against TBI [102], and SIRT1 can negatively regulate NLRP3 in vascular endothelial cells [103].

In conclusion, TBI can activate the NLRP3 inflammasome, thereby promoting release of the pro-inflammatory cytokines IL-1β and IL-18, and amplifying brain injury. Resveratrol might attenuate the inflammatory response and relieve TBI by reducing ROS production and inhibiting activation of the NLRP3 inflammasome, which prevents excessive release of pro-inflammatory cytokines. The effect of resveratrol on the NLRP3 inflammasome and ROS production might be dependent upon SIRT1 [94].

### SIRT1–NLRP3 pathway in neuroinflammation and depression

Suppression of neuroinflammation is mediated by regulation of the SIRT1-NLRP3/Nrf2 pathway (Table 1). The NLRP3 inflammasome is activated by numerous divergent invading pathogens and cellular damage (e.g., ROS, mitochondrial DNA, ATP) and subsequent excretion of pro-inflammatory cytokines (e.g., IL-18 and IL-1β) into the extracellular matrix and prolonged immunological reactions that, ultimately, result in neurotransmitter dysfunction and oxidative damage to neurons [30].

High levels of SIRT1 in the hippocampus and cortex have pivotal roles in cellular events such as aging, inflammation, homeostasis, metabolic activities, and survival [104]. Recently, SIRT1 has been linked to major depressive disorder [105]. Several studies in animal models also support the important role of SIRT1 in preventing and treating depression. Some studies have demonstrated that expression of pro-inflammatory cytokines is inhibited by SIRT1 by mediation, initiation, and progression of inflammation (e.g., deacetylating NF-κB) and, ultimately, prevents behavioral deficits (depressive and anxiety disorders) caused by chronic stress in rodents [14, 15] (Table 1). A recent study reported that increased expression of SIRT1 overcomes lipopolysaccharide-associated acute depressive-like behavior by suppression of the NLRP3 inflammasome in microglia [17]. Studies have demonstrated that anxiety-like behavior caused by brain hypoxia can be suppressed by SIRT1 via the NF-κB pathway [29].

### SIRT1-NLRP3 pathway in atherosclerosis

Activation of the NLRP3 inflammasome by extracellular metabolites has also been implicated in several other diseases, such as atherosclerosis [106]. Activation of TLRs (possibly by free fatty acids or oxidized low-density lipoprotein) and NLRP3 leads to the production of active IL-1β [107] (Table 1). IL-1β levels increase in arterial plaques, and levels of IL-1β correlate directly with disease severity [31, 32].

### SIRT1-NLRP3 pathway in liver damage

Following activation of the NLRP3 inflammasome, active caspase-1 is released from pro-caspase-1, which stimulates the release of mature IL-1β and IL-18 from its pro-form. Caspase-1 has a crucial role in exacerbating liver damage [108–111]. In addition, IL-1β stimulates the expression of other pro-inflammatory mediators and recruits neutrophils to inflamed hepatic tissue, thereby amplifying the inflammatory response [34–36].

Cucurbitacin E glucoside (CuE) has potent anti-inflammatory, immunomodulatory, and anti-tumor properties. CuE can increase the mRNA expression of SIRT1 and Nrf2 as well as its binding capacity (Table 1). Subsequently, CuE augments the mRNA expression of Nrf2-targeted genes such as NAD(P)H quinone dehydrogenase 1(NQO1), Glutamate cysteine ligase (GCL), and HO-1,and recovers their normal expression.CuE can inhibit activation of signaling of NF-κB/downstream pro-inflammatory mediators. Furthermore, CuE can attenuate the mRNA expression of NLRP3 and its associated genes [12].

## Perspective

SIRT1-NLRP3 inflammatory pathway exists in a variety of diseases, and its clinical research has become a new research point. As the well-known physicist Richard Feynman once said, “There is a pleasure in recognising old things from a new viewpoint.” These new insights into “old pathways” could give rise to a substantial increase in our understanding of the pathogenesis of inflammatory diseases, which might ultimately give rise to better treatments.

## Conclusions

**Fig. 4:**
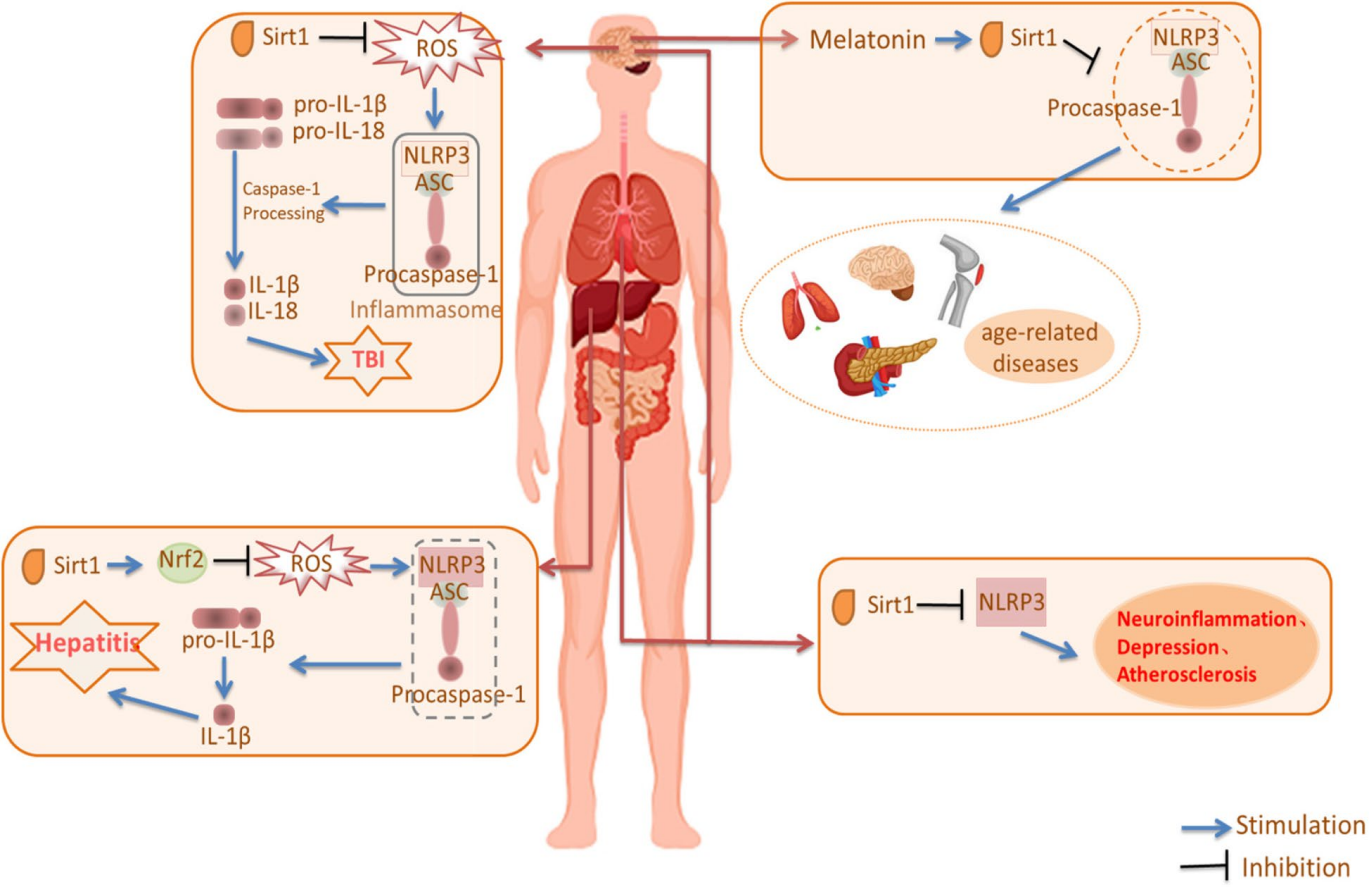
The SIRT1-NLRP3 pathway in several inflammation-related diseases. SIRT1-NLRP3 pathway influences the inflammatory response and relates to melatonin, traumatic brain injury, neuroinflammation, depression, atherosclerosis, and liver damage. (Sirt1: Silent information regulator 2 homolog 1; ROS: Reactive oxygen species; NLRP3: NACHT, LRR and PYD domains-containing protein 3; ASC: a protein; IL: Interleukin; TBI: Traumatic brain injury; Nrf2: Nuclear factor erythroid 2-related factor 2)

The SIRT1-NLRP3 pathway is closely related to the occurrence and development of several inflammation-related diseases (Fig. 4). SIRT1 can inhibit this inflammatory response through Nrf2 and NF-κB pathways by reducing the NLRP3 inflammasome. Many drugs can exert anti-inflammatory effects based on the inhibition of NLRP3 from SIRT1, including many chemical drugs and traditional Chinese medicines, such as Huaiqihuang, Ginsenoside Rg3, Omeprazole, Astragaloside IV, Salvianolic acid B and Salvianolic acid [108, 112–116].

## Data Availability

Data available on request from the authors.

## Abbreviations

SIRT1: Silent information regulator 2 homolog 1
NLRP3: NACHT, LRR and PYD domains-containing protein 3
IL: Interleukin
TNF: Tumor necrosis factor
IFN: Interferon
ROS: Reactive oxygen species
NAD: Nicotinamide adenine dinucleotide
NF-κB: Nuclear factor kappa B
Nrf2: Nuclear factor erythroid 2-related factor 2
DNA: Deoxyribonucleic acid
RNA: Ribonucleic acid
TLR: Toll-like receptor
HO: Heme oxidase
PYD: Pyrin domain
LRR: Leucine-rich repeat
NACHT: Domain present in NAIP, CIITA, HETE and TP1
ATP: Adenosine triphosphate
ADP: Adenosine diphosphate
CARD: C-terminal caspase-recruitment domain
NEK7: NIMA-related kinase 7
AIM2: Absent in melanoma 2
Wnt: Wingless type
HMGB: High mobility group box
COPD: Chronic obstructive pulmonary disease
NAMPT: Nicotinamide phosphoribosyltransferase
MT: Membrane receptor
CNS: Central nervous system
TBI: Traumatic brain injury
CuE: Cucurbitacin E glucoside
NQO1: NAD(P)H quinone dehydrogenase 1
GCL: Glutamate cysteine ligase

## References

[1] Swanson KV, Deng M, Ting JP (2019). The NLRP3 inflammasome: molecular activation and regulation to therapeutics. Nat Rev Immunol.

[2] Mangan MSJ (2018). Targeting the NLRP3 inflammasome in inflammatory diseases. Nat Rev Drug Discov.

[3] McGettrick AF, O'Neill LA (2013). How metabolism generates signals during innate immunity and inflammation. J Biol Chem.

[4] Manji GA (2002). PYPAF1, a PYRIN-containing Apaf1-like protein that assembles with ASC and regulates activation of NF-kappa B. J Biol Chem.

[5] Franchi L (2009). Function of Nod-like receptors in microbial recognition and host defense. Immunol Rev.

[6] Martinon F, Burns K, Tschopp J (2002). The inflammasome: a molecular platform triggering activation of inflammatory caspases and processing of proIL-beta. Mol Cell.

[7] Dinarello CA (2009). Immunological and inflammatory functions of the interleukin-1 family. Annu Rev Immunol.

[8] Kelley N (2019). The NLRP3 inflammasome: an overview of mechanisms of activation and regulation. Int J Mol Sci.

[9] Hoffman HM (2001). Mutation of a new gene encoding a putative pyrin-like protein causes familial cold autoinflammatory syndrome and muckle-wells syndrome. Nat Genet.

[10] Nogueiras R (2012). Sirtuin 1 and sirtuin 3: physiological modulators of metabolism. Physiol Rev.

[11] Chen C (2020). SIRT1 and aging related signaling pathways. Mech Ageing Dev.

[12] Mohamed GA (2022). Cucurbitacin E glucoside alleviates concanavalin A-induced hepatitis through enhancing SIRT1/Nrf2/HO-1 and inhibiting NF-kB/NLRP3 signaling pathways. J Ethnopharmacol.

[13] Wei L (2022). The SIRT1-HMGB1 axis: Therapeutic potential to ameliorate inflammatory responses and tumor occurrence. Front Cell Develop Biol.

[14] Abe-Higuchi N (2016). Hippocampal sirtuin 1 signaling mediates depression-like behavior. Biol Psychiatry.

[15] Lu G (2018). Role and possible mechanisms of sirt1 in depression. Oxid Med Cell Longev.

[16] Singh CK (2018). The role of sirtuins in antioxidant and redox signaling. Antioxid Redox Signal.

[17] Arioz BI (2019). Melatonin attenuates lps-induced acute depressive-like behaviors and microglial NLRP3 inflammasome activation through the SIRT1/Nrf2 pathway. Front Immunol.

[18] Peng XP (2019). The protective effect of oleanolic acid on NMDA-induced MLE-12 cells apoptosis and lung injury in mice by activating SIRT1 and reducing NF-kappaB acetylation. Int Immunopharmacol.

[19] Mohamed GA (2021). Terretonin as a new protective agent against sepsis-induced acute lung injury: impact on SIRT1/Nrf2/NF-kappaBp65/NLRP3 signaling. Biology (Basel).

[20] Yamamoto M, Kensler TW, Motohashi H (2018). The KEAP1-NRF2 system: a thiol-based sensor-effector apparatus for maintaining redox homeostasis. Physiol Rev.

[21] Wardyn JD, Ponsford AH, Sanderson CM (2015). Dissecting molecular cross-talk between Nrf2 and NF-kappaB response pathways. Biochem Soc Trans.

[22] Sivandzade F (2019). NRF2 and NF-қB interplay in cerebrovascular and neurodegenerative disorders: Molecular mechanisms and possible therapeutic approaches. Redox Biol.

[23] Ahmed SM (2017). Nrf2 signaling pathway: pivotal roles in inflammation. Biochim Biophys Acta Mol Basis Dis.

[24] Lee H, Choi YK (2018). Regenerative effects of heme oxygenase metabolites on neuroinflammatory diseases. Int J Mol Sci.

[25] Hardeland R (2018). Melatonin and inflammation-story of a double-edged blade. J Pineal Res.

[26] Liu Y (2014). TXNIP mediates NLRP3 inflammasome activation in cardiac microvascular endothelial cells as a novel mechanism in myocardial ischemia/reperfusion injury. Basic Res Cardiol.

[27] Yin N (2016). Isoflurane attenuates lipopolysaccharide-induced acute lung injury by inhibiting ROS-mediated NLRP3 inflammasome activation. Am J Transl Res.

[28] Tang BL (2016). Sirt1 and the Mitochondria. Mol Cells.

[29] Fan J (2018). SIRT1 mediates Apelin-13 in ameliorating chronic normobaric hypoxia-induced anxiety-like behavior by suppressing NF-kappaB pathway in mice hippocampus. Neuroscience.

[30] Heneka MT, McManus RM, Latz E (2018). Inflammasome signalling in brain function and neurodegenerative disease. Nat Rev Neurosci.

[31] Galea J (1996). Interleukin-1 beta in coronary arteries of patients with ischemic heart disease. Arterioscler Thromb Vasc Biol.

[32] Moyer CF (1991). Synthesis of IL-1 alpha and IL-1 beta by arterial cells in atherosclerosis. Am J Pathol.

[33] Peng Z (2018). Melatonin attenuates airway inflammation via SIRT1 dependent inhibition of NLRP3 inflammasome and IL-1beta in rats with COPD. Int Immunopharmacol.

[34] Wree A (2014). NLRP3 inflammasome activation results in hepatocyte pyroptosis, liver inflammation, and fibrosis in mice. Hepatology.

[35] Guo S (2015). The NLRP3 inflammasome and IL-1beta accelerate immunologically mediated pathology in experimental viral fulminant hepatitis. PLoS Pathog.

[36] Elsaed WM (2019). Amygdalin (Vitamin B17) pretreatment attenuates experimentally induced acute autoimmune hepatitis through reduction of CD4+ cell infiltration. Ann Anat.

[37] Song R (2016). Melatonin promotes diabetic wound healing in vitro by regulating keratinocyte activity. Am J Transl Res.

[38] Duncan JA (2007). Cryopyrin/NALP3 binds ATP/dATP, is an ATPase, and requires ATP binding to mediate inflammatory signaling. Proc Natl Acad Sci U S A.

[39] Cai X (2014). Prion-like polymerization underlies signal transduction in antiviral immune defense and inflammasome activation. Cell.

[40] Lu A (2014). Unified polymerization mechanism for the assembly of ASC-dependent inflammasomes. Cell.

[41] Schmidt FI (2016). A single domain antibody fragment that recognizes the adaptor ASC defines the role of ASC domains in inflammasome assembly. J Exp Med.

[42] Boucher D (2018). Caspase-1 self-cleavage is an intrinsic mechanism to terminate inflammasome activity. J Exp Med.

[43] Schmid-Burgk JL (2016). A genome-wide CRISPR (Clustered Regularly Interspaced Short Palindromic Repeats) screen identifies NEK7 as an essential component of NLRP3 inflammasome activation. J Biol Chem.

[44] He Y (2016). NEK7 is an essential mediator of NLRP3 activation downstream of potassium efflux. Nature.

[45] Shi H (2016). NLRP3 activation and mitosis are mutually exclusive events coordinated by NEK7, a new inflammasome component. Nat Immunol.

[46] Jiang N (2022). Ginsenosides Rb1 Attenuates Chronic Social Defeat Stress-Induced Depressive Behavior via Regulation of SIRT1-NLRP3/Nrf2 Pathways. Front Nutr.

[47] Bauernfeind FG (2009). Cutting edge: NF-kappaB activating pattern recognition and cytokine receptors license NLRP3 inflammasome activation by regulating NLRP3 expression. J Immunol.

[48] Netea MG (2009). Differential requirement for the activation of the inflammasome for processing and release of IL-1beta in monocytes and macrophages. Blood.

[49] Latz E, Xiao TS, Stutz A (2013). Activation and regulation of the inflammasomes. Nat Rev Immunol.

[50] Yeung F (2004). Modulation of NF-kappaB-dependent transcription and cell survival by the SIRT1 deacetylase. EMBO J.

[51] Ashrafizadeh M (2021). Anti-inflammatory activity of melatonin: a focus on the role of NLRP3 inflammasome. Inflammation.

[52] Tordjman S (2017). Melatonin: pharmacology, functions and therapeutic benefits. Curr Neuropharmacol.

[53] Zhou H, et al. Melatonin suppresses platelet activation and function against cardiac ischemia/reperfusion injury via PPARgamma/FUNDC1/mitophagy pathways. J Pineal Res. 2017; 63(4):e12438. 10.1111/jpi.12438 28749565

[54] Habtemariam S (2017). Melatonin and respiratory diseases: a review. Curr Top Med Chem.

[55] Zhang Y (2016). Melatonin alleviates acute lung injury through inhibiting the NLRP3 inflammasome. J Pineal Res.

[56] Hardeland R (2019). Aging, melatonin, and the pro- and anti-inflammatory networks. Int J Mol Sci.

[57] Rabadi MM (2015). High-mobility group box 1 is a novel deacetylation target of Sirtuin1. Kidney Int.

[58] Hwang JS (2015). Deacetylation-mediated interaction of SIRT1-HMGB1 improves survival in a mouse model of endotoxemia. Sci Rep.

[59] Yuan Y (2018). SIRT1 attenuates murine allergic rhinitis by downregulated HMGB 1/TLR4 pathway. Scand J Immunol.

[60] Rickenbacher A (2014). Fasting protects liver from ischemic injury through Sirt1-mediated downregulation of circulating HMGB1 in mice. J Hepatol.

[61] Xu W (2014). Novel role of resveratrol: suppression of high-mobility group protein box 1 nucleocytoplasmic translocation by the upregulation of sirtuin 1 in sepsis-induced liver injury. Shock.

[62] Hwang JS (2014). Ligand-activated peroxisome proliferator-activated receptor-delta and -gamma inhibit lipopolysaccharide-primed release of high mobility group box 1 through upregulation of SIRT1. Cell Death Dis.

[63] Yin Y (2017). SIRT1 inhibits releases of HMGB1 and HSP70 from human umbilical vein endothelial cells caused by IL-6 and the serum from a preeclampsia patient and protects the cells from death. Biomed Pharmacother.

[64] Chen X (2017). Omega-3 polyunsaturated fatty acid supplementation attenuates microglial-induced inflammation by inhibiting the HMGB1/TLR4/NF-kappaB pathway following experimental traumatic brain injury. J Neuroinflammation.

[65] Hwang JS (2018). Formononetin inhibits lipopolysaccharide-induced release of high mobility group box 1 by upregulating SIRT1 in a PPARdelta-dependent manner. PeerJ.

[66] Schaper F (2016). High mobility group box 1 skews macrophage polarization and negatively influences phagocytosis of apoptotic cells. Rheumatology(Oxford).

[67] Karuppagounder V (2016). Modulation of Macrophage Polarization and HMGB1-TLR2/TLR4 cascade plays a crucial role for cardiac remodeling in senescence-accelerated prone mice. PLoS ONE.

[68] Son M (2016). C1q and HMGB1 reciprocally regulate human macrophage polarization. Blood.

[69] Jiang Y (2018). HMGB1 silencing in macrophages prevented their functional skewing and ameliorated EAM development: Nuclear HMGB1 may be a checkpoint molecule of macrophage reprogramming. Int Immunopharmacol.

[70] Gao T (2018). Inhibition of HMGB1 mediates neuroprotection of traumatic brain injury by modulating the microglia/macrophage polarization. Biochem Biophys Res Commun.

[71] Hwang JW (2013). Redox regulation of SIRT1 in inflammation and cellular senescence. Free Radic Biol Med.

[72] Li Y (2016). SIRT1 inhibits inflammatory response partly through regulation of NLRP3 inflammasome in vascular endothelial cells. Mol Immunol.

[73] Eapen MS (2017). Airway inflammation in chronic obstructive pulmonary disease (COPD): a true paradox. Expert Rev Respir Med.

[74] Eltom S (2014). Role of the inflammasome-caspase1/11-IL-1/18 axis in cigarette smoke driven airway inflammation: an insight into the pathogenesis of COPD. PLoS ONE.

[75] Fu JJ (2015). Airway IL-1beta and systemic inflammation as predictors of future exacerbation risk in asthma and COPD. Chest.

[76] Colarusso C (2017). Role of the inflammasome in chronic obstructive pulmonary disease (COPD). Oncotarget.

[77] Yanagisawa S (2017). Decreased serum Sirtuin-1 in COPD. Chest.

[78] Yao H (2015). Disruption of Sirtuin 1-mediated control of circadian molecular clock and inflammation in chronic obstructive pulmonary disease. Am J Respir Cell Mol Biol.

[79] Zhang L (2015). Resveratrol exerts an anti-apoptotic effect on human bronchial epithelial cells undergoing cigarette smoke exposure. Mol Med Rep.

[80] Bai XZ (2016). Melatonin prevents acute kidney injury in severely burned rats via the activation of SIRT1. Sci Rep.

[81] Han D (2016). Melatonin facilitates adipose-derived mesenchymal stem cells to repair the murine infarcted heart via the SIRT1 signaling pathway. J Pineal Res.

[82] Xu L (2018). Melatonin suppresses estrogen deficiency-induced osteoporosis and promotes osteoblastogenesis by inactivating the NLRP3 inflammasome. Calcif Tissue Int.

[83] Amstrup AK (2013). Melatonin and the skeleton. Osteoporos Int.

[84] Aravamudhan A (2013). Osteoinductive small molecules: growth factor alternatives for bone tissue engineering. Curr Pharm Des.

[85] Luchetti F (2014). Melatonin regulates mesenchymal stem cell differentiation: a review. J Pineal Res.

[86] Vriend J, Reiter RJ (2016). Melatonin, bone regulation and the ubiquitin-proteasome connection: a review. Life Sci.

[87] Korkmaz A, Rosales-Corral S, Reiter RJ (2012). Gene regulation by melatonin linked to epigenetic phenomena. Gene.

[88] Ortiz F (2015). Melatonin blunts the mitochondrial/NLRP3 connection and protects against radiation-induced oral mucositis. J Pineal Res.

[89] Fernandez-Gil B (2017). Melatonin protects rats from radiotherapy-induced small intestine toxicity. PLoS ONE.

[90] Garcia JA (2015). Disruption of the NF-kappaB/NLRP3 connection by melatonin requires retinoid-related orphan receptor-alpha and blocks the septic response in mice. FASEB J.

[91] Volt H (2016). Same molecule but different expression: aging and sepsis trigger NLRP3 inflammasome activation, a target of melatonin. J Pineal Res.

[92] Liu Z, et al. Melatonin alleviates inflammasome-induced pyroptosis through inhibiting NF-kappaB/GSDMD signal in mice adipose tissue. J Pineal Res. 2017;63(1):e12414. 10.1111/jpi.12414 28398673

[93] Mayo JC, et al. Melatonin and sirtuins: A "not-so unexpected" relationship. J Pineal Res. 2017;62(2):e12391. 10.1111/jpi.12391 28109165

[94] Zou P (2018). Resveratrol pretreatment attenuates traumatic brain injury in rats by suppressing NLRP3 inflammasome activation via SIRT1. Mol Med Rep.

[95] Zhang Y (2020). XingNaoJing injection ameliorates cerebral ischaemia/reperfusion injury via SIRT1-mediated inflammatory response inhibition. Pharm Biol.

[96] Qu X (2019). XingNaoJing injections protect against cerebral ischemia/reperfusion injury and alleviate blood-brain barrier disruption in rats, through an underlying mechanism of NLRP3 inflammasomes suppression. Chin J Nat Med.

[97] Minutoli L (2016). ROS-Mediated NLRP3 inflammasome activation in brain, heart, kidney, and testis ischemia/reperfusion injury. Oxid Med Cell Longev.

[98] Hanslik KL, Ulland TK (2020). The role of microglia and the nlrp3 inflammasome in alzheimer's disease. Front Neurol.

[99] Li L, Liu Y (2015). Aging-related gene signature regulated by Nlrp3 predicts glioma progression. Am J Cancer Res.

[100] Liu HD (2013). Expression of the NLRP3 inflammasome in cerebral cortex after traumatic brain injury in a rat model. Neurochem Res.

[101] Martin A (2015). Role of SIRT1 in autoimmune demyelination and neurodegeneration. Immunol Res.

[102] Zhao Y (2012). Interactions between SIRT1 and MAPK/ERK regulate neuronal apoptosis induced by traumatic brain injury in vitro and in vivo. Exp Neurol.

[103] Li Y (2017). Negative regulation of NLRP3 inflammasome by SIRT1 in vascular endothelial cells. Immunobiology.

[104] Tang X (2020). Resveratrol mitigates sevoflurane-induced neurotoxicity by the SIRT1-dependent regulation of bdnf expression in developing mice. Oxid Med Cell Longev.

[105] Lei Y (2020). SIRT1 in forebrain excitatory neurons produces sexually dimorphic effects on depression-related behaviors and modulates neuronal excitability and synaptic transmission in the medial prefrontal cortex. Mol Psychiatry.

[106] Zhai J (2020). Calycosin ameliorates doxorubicin-induced cardiotoxicity by suppressing oxidative stress and inflammation via the sirtuin 1-NOD-like receptor protein 3 pathway. Phytotherapy research.

[107] Tannahill GM, O'Neill LA (2011). The emerging role of metabolic regulation in the functioning of Toll-like receptors and the NOD-like receptor Nlrp3. FEBS Lett.

[108] Zhai J (2021). Ginsenoside Rg3 attenuates cisplatin-induced kidney injury through inhibition of apoptosis and autophagy-inhibited NLRP3. J Biochem Mol Toxicol.

[109] Zhang Y (2019). Quercetin protected against isoniazide-induced HepG2 cell apoptosis by activating the SIRT1/ERK pathway. J Biochem Mol Toxicol.

[110] Qu X, et al. Autophagy inhibition-enhanced assembly of the NLRP3 inflammasome is associated with cisplatin-induced acute injury to the liver and kidneys in rats. J Biochem Mol Toxicol. 2018;e22208. 10.1002/jbt.22228 30291731

[111] Qu X (2018). Dysregulation of BSEP and MRP2 May Play an Important Role in Isoniazid-Induced Liver Injury via the SIRT1/FXR Pathway in Rats and HepG2 Cells. Biol Pharm Bull.

[112] Zhang Y (2021). Huaiqihuang (HQH) granule alleviates cyclophosphamide-induced nephrotoxicity via suppressing the MAPK/NF-κB pathway and NLRP3 inflammasome activation. Pharm Biol.

[113] Gao H (2020). Omeprazole attenuates cisplatin-induced kidney injury through suppression of the TLR4/NF-κB/NLRP3 signaling pathway. Toxicology.

[114] Zhai J (2019). Salvianolic Acid B Attenuates Apoptosis of HUVEC Cells Treated with High Glucose or High Fat via Sirt1 Activation. Evidence-based Complement Alternat Med.

[115] Qu X (2019). Astragaloside IV protects against cisplatin-induced liver and kidney injury via autophagy-mediated inhibition of NLRP3 in rats. J Toxicol Sci.

[116] Zhai J (2019). Salvianolic acid inhibits the effects of high glucose on vascular endothelial dysfunction by modulating the Sirt1-eNOS pathway. J Biochem Mol Toxicol.

